# Supplementary material for: Octahedral Molybdenum Cluster-Based Nanomaterials for Potential Photodynamic Therapy
Source: Nanomaterials (Basel). 2022 Sep 26;12(19):3350. doi: 10.3390/nano12193350 (PMC9565569; doi:10.3390/nano12193350)
Supplement: Supplementary file 1 [file nanomaterials-12-03350-s001.zip › nanomaterials-1898642-supplementary.pdf]

nanomaterials

MDPI

Supplementary Materials

Octahedral Molybdenum Cluster-Based Nanomaterials for Potential Photodynamic Therapy

Marina Rodrigues Tavares [1], Kaplan Kirakci [2], Nikolay Kotov [1], Michal Pechar [1], Kamil Lang [2], Robert Pola [1,*] and Tomáš Etrych [1]

[1] Institute of Macromolecular Chemistry of the Czech Academy of Sciences, Heyrovského náměstí 2, 162 06 Prague 6, Czech Republic

[2] Institute of Inorganic Chemistry of the Czech Academy of Sciences, 250 68 Husinec-Řež 1001, Czech Republic

* Correspondence: pola@imc.cas.cz; Tel.: +420–296–809–216

Table of Contents

S1. Synthesis of Copolymer Precursors **P1–P5**

S2. Nuclear Magnetic Resonance (NMR) Spectroscopy

S1. Synthesis of Copolymer Precursors P1-P5

*S1.1. Synthesis of Polymer Precursor poly(HPMA-co-MA-AP-COOH)—**P1**.*

The polymerization of HPMA and MA-AP-TT was carried out in a mixture of *t*-butanol/DMA (8/2) at 70 °C for 16 h, using AIBN as initiator. The molar ratio of monomers/CTA-AIBN/AIBN was 350/2/1 and the ratio of monomers HPMA and MA-AP-TT was 98/2. The detailed synthetic procedure is as follows: HPMA (250 mg, 1.75 mmol) was dissolved in *t*-butanol (1.58 mL) and mixed with a solution of MA-AP-TT (9.7 mg, 37 μmol), AIBN as initiator (1.43 mg, 5.1 μmol), and CTA-AIBN (2.25 mg, 10.2 μmol) in DMA (396 μL)—the mixture corresponds to 0.9 M solution of monomers. The reaction mixture was poured into a glass ampoule, bubbled with argon, and sealed. After 16 h in a thermostat-controlled water bath at 70°C, the ampoule was cooled, and isolation was performed by dropping the solution into a mixture of dry acetone and dry diethyl ether (2/1; 100 mL) as non-solvent. After centrifugation at 7800 RPM for 5 min, the polymer precipitate was filtered off and purified by reprecipitation from methanol (2.5 mL) into the non-solvent mixture. The copolymer was filtered off and dried under vacuum (161 mg, 64 %). For the removal of the dithiobenzoate group, 160 mg of the copolymer and AIBN (40 mg) were dissolved in DMA (2 mL), poured into a glass ampoule, bubbled with argon, and sealed. After 3 h in a thermostat-controlled water bath at 80 °C, the isolation and purification procedures were performed similarly as before. The sample was dried under vacuum, affording the precursor **P0a**, poly(HPMA-*co*-MA-AP-TT) (142 mg, 89 %). Hydrolysis of TT groups was performed by dissolution of **P0a** (70 mg, 9.6 μmol of TT groups) in 1.4 mL of phosphate buffer (pH 8.0). After stirring overnight at room temperature, the reaction mixture was purified by gel filtration using a Sephadex G-25 with water as eluent and UV detection. The hydrolysis of TT groups was monitored by high-performance liquid chromatography (HPLC) and the polymer fraction was freeze-dried, affording the final precursor poly(HPMA-*co*-MA-AP-COOH) (66 mg, 94 %). For characterization of the polymer precursor **P1**, see Table 1 of the main text.

*S1.2. Synthesis of Polymer Precursor poly(HPMA-co-APMA)—**P2**.*

The polymerization of poly(HPMA-*co*-APMA-Boc) was carried out in a mixture of water/dioxane (2/1) at 70 °C for 7 h, using ACVA as initiator. The molar ratio of monomers/CTA-ACVA/ACVA was 350/2/1 and the ratio of monomers HPMA and APMA-Boc was 94/6. The detailed synthetic procedure is as follows: HPMA (0.5 g, 3.49 mmol) was

dissolved in water (2.48 mL) and mixed with a solution of APMA-Boc (51 mg, 0.22 mmol), ACVA as initiator (2.97 mg, 10.6 μmol) and CTA-ACVA (6.2 mg, 21.2 μmol) in 1,4-dioxane (1.24 mL)—the mixture results in 1 M solution of monomers. The reaction mixture was poured into a glass ampoule, bubbled with argon, and sealed. After 7 h in a thermostat-controlled water bath at 70 °C, the ampoule was cooled, and isolation was performed by dropping the solution into 100 mL of acetone. After centrifugation at 7800 RPM for 5 min, the polymer precipitate was filtered off and purified by reprecipitation from methanol (4 mL) into a mixture of acetone and diethyl ether (2/1, 100 mL). The copolymer was filtered off and dried under vacuum (0.4 g, 72 %). The procedure for the removal of the dithiobenzoate end group was analogous to the one described for polymer precursor **P1**, however, here 400 mg of the copolymer and 60 mg of AIBN were dissolved in 4 mL of DMA. The sample was dried under vaccum, affording the copolymer still containing Boc-protected amine groups (370 mg, 92 %). Boc group removal was performed by dissolving the copolymer in Q-H_2O (10 wt. %) and placing the ampoule into a thermostat-controlled oil bath at 150 °C for 1 h. After purification in Sephadex G-25 with water as eluent and UV detection, the polymer was freeze-dried, affording the final precursor poly(HPMA-*co*-APMA) (0.27 g, 73 %). For characterization of the polymer precursor **P2**, see Table 1 of the main text.

*S1.3. Synthesis of Polymer Precursor poly(HPMA-co-MA-AH-cholesterol)—**P3**.*

The polymerization of HPMA and MA-AH-cholesterol was carried out in a mixture of *t*-butanol/DMA (9/1) at 40 °C for 16 h, using V-70 as initiator. The molar ratio of monomers/CTA-AIBN/V-70 was 500/2/1 and the ratio of monomers HPMA and MA-AH-cholesterol was 97.7/2.3. The detailed synthetic procedure is as follows: HPMA (244.8 mg, 1.71 mmol), MA-AH-cholesterol (22.8 mg, 0.04 mmol), and CTA-AIBN (1.55 mg, 7 μmol) were dissolved in *t*-butanol (2.25 mL) and mixed with a solution of V-70 as initiator (1.08 mg, 3.5 μmol) in DMA (0.25 mL)—the mixture corresponds to 0.7 M solution of monomers. The reaction mixture was poured into a glass ampoule, bubbled with argon, and sealed. After 16 h in a thermostat-controlled water bath at 40°C, the ampoule was cooled and isolation was performed by dropping the solution into a mixture of dry acetone and dry diethyl ether (2/1; 100 mL) as non-solvent. After centrifugation at 7800 RPM for 5 min, the polymer precipitate was filtered off and purified by reprecipitation from methanol (3 mL) into the non-solvent mixture. The copolymer was filtered off and dried under vacuum (173 mg, 65 %). The procedure for the removal of the dithiobenzoate end group was analogous to the one described for polymer precursor **P1**, however, here 172 mg of the copolymer and 26 mg of AIBN were dissolved in 1.7 mL of DMA. The sample was dried under vaccum, affording the precursor poly(HPMA-*co*-MA-AH-cholesterol) (145 mg, 84 %). For characterization of the polymer precursor **P3**, see Table 1 of the main text.

*S1.4. Synthesis of Polymer Precursor poly(HPMA-co-MA-AH-cholesterol-co-APMA)—**P4**.*

The polymerization of HPMA, APMA-Boc and MA-AH-cholesterol was carried out in a mixture of *t*-butanol/DMA (9/1) at 40 °C for 16 h, using V-70 as initiator. The molar ratio of monomers/CTA-AIBN/V-70 was 350/2/1 and the ratio of monomers HPMA, APMA-Boc, and MA-AH-cholesterol was 94/4/2. The detailed synthetic procedure is as follows: HPMA (188.4 mg, 1.32 mmol), MA-AH-cholesterol (15.9 mg, 0.028 mmol), and CTA-AIBN (1.77 mg, 8 μmol) were dissolved in *t*-butanol (1.8 mL) and mixed with a solution of APMA-Boc (12.9 mg, 0.056 mmol) and V-70 (1.23 mg, 4 μmol) in DMA (0.2 mL)—the mixture corresponds to 0.7 M solution of monomers. The reaction parameters, isolation, and purification were analogous to the procedure used for **P3**, affording the polymer with CTA end (126.1 mg, 58 %). The procedure for the removal of the dithiobenzoate end group was analogous to the one described for polymer precursor **P1**, however, here 125 mg of the copolymer and 20 mg of AIBN were dissolved in 1.4 mL of DMA. The sample was dried under vaccum,

affording the copolymer still containing Boc-protected amine groups (110.2 mg, 88 %). Boc group removal was performed by dissolving the copolymer in Q-H_2O (10 wt. %) and placing the ampoule into a thermostat-controlled oil bath at 150 °C for 1 h. After purification in Sephadex G-25 with water as eluent and UV detection, the polymer was freeze-dried, affording the final precursor poly(HPMA-*co*-MA-AH-cholesterol-*co*-APMA) (82.4 mg, 75 %). For characterization of the polymer precursor **P4**, see Table 1 of the main text.

*S1.5. Synthesis of Polymer Precursor poly(HPMA-co-MA-AP-DBCO)—**P5***

The polymerization of HPMA and MA-AP-TT was carried out in a mixture of *t*-butanol/DMA (8/2) at 40 °C for 16 h, using V-70 as initiator. The ratio of monomers HPMA and MA-AP-TT was 90/10 and the molar ratio of monomers/CTA-AIBN/V-70 was 550/2/1. After CTA end group removal, the polymer precursor bearing 8 mol. % of DBCO groups was prepared via aminolysis of TT groups of the polymer precursor poly(HPMA-*co*-MA-AP-TT) with the amine-functionalized DBCO in DMA. The detailed synthetic procedure is as follows: HPMA (500 mg, 3.49 mmol) was dissolved in *t*-butanol (4.9 mL) and mixed with a solution of MA-AP-TT (100 mg, 0.39 mmol), V-70 (2.1 mg, 6.8 μmol), and CTA-AIBN (3.0 mg, 13.6 μmol) in DMA (550 μL) – the mixture corresponds to 0.7 M solution of monomers. The reaction parameters, isolation and purification were analogous to the procedure used for **P3**, affording the polymer with CTA end (450 mg, 75 %). The procedure for the removal of the dithiobenzoate end group was analogous to the one described for polymer precursor **P1**, however, here 449 mg of the copolymer and 90 mg of AIBN were dissolved in 5 mL of DMA. Sample was dried under vaccum, affording the precursor **P0b**, poly(HPMA-*co*-MA-β-AP-TT) (380 mg, 85 %). Aminolysis of TT groups was performed by the reaction of **P0b** with the amine-functionalized spacer DBCO, affording the final polymer precursor bearing dibenzocyclooctyne (DBCO) groups poly(HPMA-*co*-MA-AP-DBCO). For this, DBCO-amine (55.1 mg, 0.20 mmol) was dissolved in DMA (500 μl) then added to a solution of precursor poly(HPMA-*co*-MA-AP-TT) (380 mg, 0.25 mmol of TT groups) in DMA (3.5 mL) at room temperature under stirring. DIPEA (35 μL, 0.20 mmol) was slowly dropped and, after 30 min stirring, unreacted TT groups were quenched with 1-aminopropan-2-ol (10 μL, 0.13 mmol). Purification of the reaction mixture was performed using Sephadex LH-20 column with methanol elution and UV detection. The polymer-containing fraction was concentrated in vacuum, diluted in methanol (4 mL) and the final polymer precursor was isolated by precipitation into acetone and diethyl ether (2/1; 100 mL). The sample was filtrated and dried under vacuum to yield **P5**, poly(HPMA-*co*-MA-AP-DBCO) (386 mg, 93 %). Attachment of DBCO groups and the hydrolysis of remaining TT groups were monitored by HPLC. For characterization of the polymer precursor **P5**, see Table 1 of the main text.

Detailed structures of copolymer precursors **P1**–**P5** are shown in Figure 2, and their physico-chemical characterization is given in Table 1 of the main text.

S2. Nuclear Magnetic Resonance (NMR) Spectroscopy

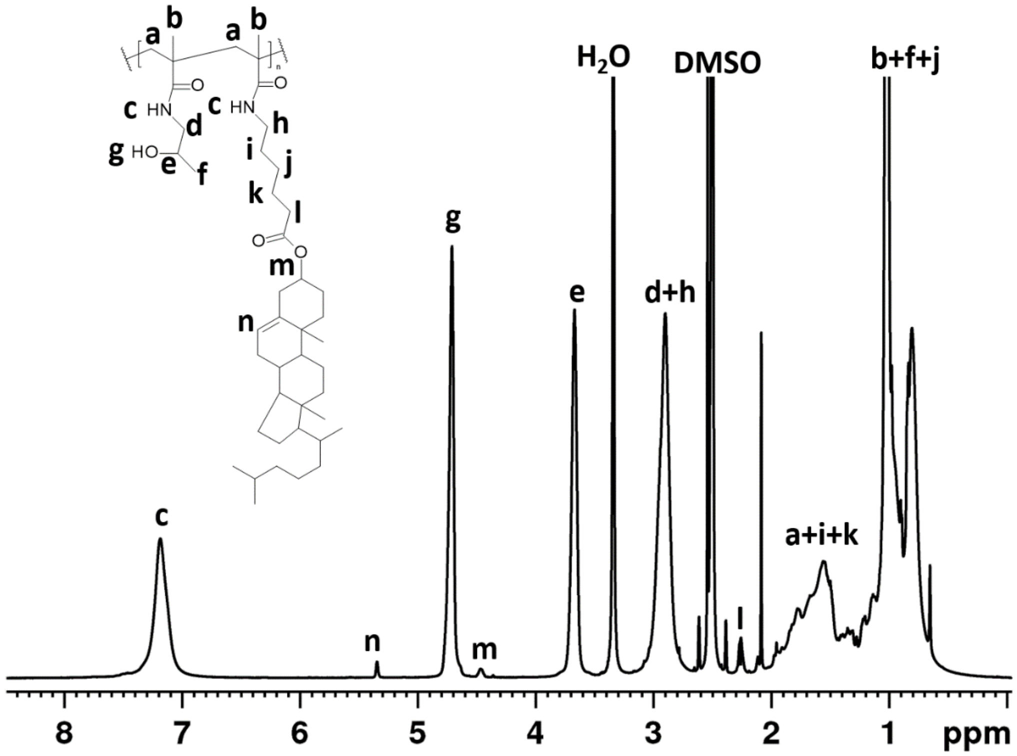

Figure S1. ^{1}H NMR spectrum of polymer precursor **P3** (600.23 MHz for ^{1}H, DMSO-d_6, 22 °C).

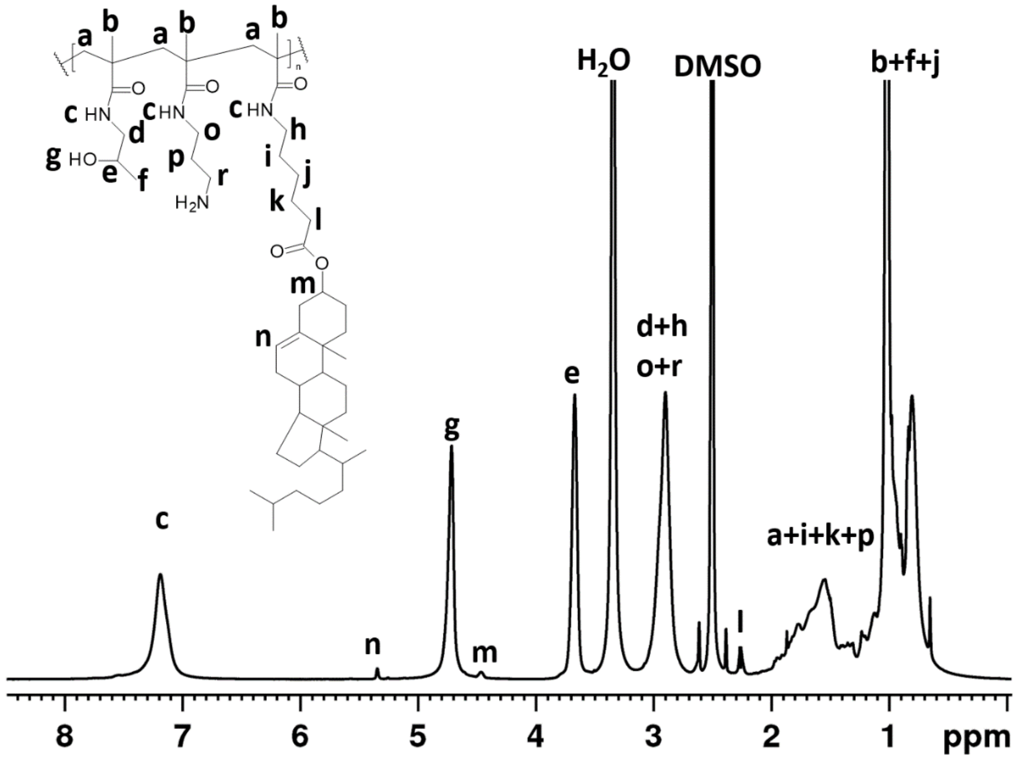

Figure S2. ^{1}H NMR spectrum of polymer precursor **P4** (600.23 MHz for ^{1}H, DMSO-d_6, 22 °C).

Abbreviations

ACVA, 4,4′-azo*bis*(4-cyanopentanoic acid); AIBN, 2,2′-azo*bis*isobutyronitrile; APMA-Boc, *N*-(3-*tert*-butoxycarbonyl-aminopropyl)methacrylamide; CTA-ACVA, 4-cyano-4-(thiobenzoylthio)pentanoic acid; CTA-AIBN, 2-cyanopropan-2-yl dithiobenzoate; DBCO, dibenzocyclooctyne; DIPEA, *N*,*N*-diisopropylethylamine; DMA, *N*,*N*-dimethylacetamide; HPMA, *N*-(2-hydroxypropyl)methacrylamide; MA-AH-cholesterol, Cholest-5en-3*β*-yl 6-methacrylamido hexanoate; MA-AP-TT, *N*-methacryloyl-*β*-alanine thiazolidine-2-thione; *t*-Boc group, *tert*-butoxycarbonyl group; TT group, thiazolidine-2-thione group; V-70, 2,2'-azo*bis*(4-methoxy-2,4-dimethylvaleronitrile).
